# From ‘traditional’ remedies to ‘modern’ supplements: a systematic review and meta-analysis of pharmaceutical adulteration in weight-loss natural products

**DOI:** 10.3389/fphar.2025.1594975

**Published:** 2025-05-15

**Authors:** Dang Thuc Anh Phan, Chuenjid Kongkaew, Michael Heinrich, Thi Cam Minh Dao, Thi Ha Vo

**Affiliations:** ^1^ Research Centre for Safety and Quality in Health, Faculty of Pharmaceutical Sciences, Naresuan University, Phitsanulok, Thailand; ^2^ Faculty of Pharmacy, University of Medicine and Pharmacy, Hue University, Hue, Vietnam; ^3^ Research Group Pharmacognosy and Phytotherapy, UCL School of Pharmacy, University College London, London, United Kingdom; ^4^ Department of Pharmaceutical Sciences and Chinese Medicine Resources, Chinese Medicine Research Center, College of Chinese Medicine, Taichung, Taiwan; ^5^ Department of Pharmacy, Nguyen Tri Phuong Hospital, Ho Chi Minh, Vietnam; ^6^ Faculty of Pharmacy, Pham Ngoc Thach University of Medicine, Ho Chi Minh, Vietnam

**Keywords:** herbal medicines, natural products, adulteration, synthetic pharmaceuticals, ethnopharmacology

## Abstract

**Introduction:**

The World Health Organization has identified obesity as an escalating public health concern affecting millions globally, contributing to the increasing demand for anti-obesity supplements. Traditional medicinal systems, such as Ayurveda and Traditional Chinese Medicine, have historically incorporated botanicals for weight management within a holistic therapeutic framework. However, the widespread commercialization of herbal weight-loss products has resulted in misrepresentation of traditional knowledge and the frequent adulteration of these formulations with synthetic pharmaceuticals to enhance their effectiveness. This phenomenon not only raises ethical concerns regarding the exploitation of traditional medicine but also presents significant health risks to consumers.

**Objectives:**

In order to develop a longer-term strategy to overcome the challenges of poor quality and adulterated products making medical claims, this study aims to (1) estimate the prevalence of pharmaceutical adulteration in weight-loss natural products; and (2) examine the characteristics of such adulterations.

**Methods:**

A systematic search of PubMed, CINAHL, and Google Scholar was conducted to identify relevant studies up to July 2024. The Der Simonian-Laird random-effects model was used for data pooling. Subgroup analyses and a meta-regression model were utilized to explore potential sources of heterogeneity. The quality of the included studies was assessed using the Toxicological Data Reliability Assessment Tool (ToxRTool).

**Results:**

A total of 26 studies qualified for the systematic review, while 22 studies were included in the meta-analysis. The estimated prevalence of synthetic adulteration exhibited significant variability, ranging from 0% to 100%, with an overall pooled median estimate of 37.5% (Interquartile range (IQR) 25.9%–49.6%). The prevalence of sibutramine adulteration was found to have a median rate of 21.8% (IQR 11.9%–33.5%). The subgroup analysis revealed a high prevalence of synthetic adulteration in Europe, particularly during the years 2012–2014, with a notable occurrence in samples collected from local markets.

**Conclusion:**

This systematic review and meta-analysis highlights the high prevalence of intentional adulteration in weight-loss natural products, with sibutramine as the most common adulterant. To protect public health and market integrity, a global framework is needed, emphasizing harmonized regulations, international collaboration, and public awareness. Future research should assess long-term health effects to ensure sustainable and safe healthcare solutions worldwide.

## 1 Introduction

Overweight and obesity are well-established risk factors for multiple chronic diseases (Fruh, 2017; Guglielmi, 2025). The World Health Organization has recognized obesity as a growing global public health concern, disproportionately affecting populations in developed Western countries (World Health Organization, 2024). Traditional medical systems across various cultures have historically employed medicinal plants for weight management, typically within the framework of integrative health rather than as rapid weight-loss interventions (Chandrasekaran et al., 2012). It is often linked to managing ‘appetite’ and ‘hunger’. Traditional medical systems such as Ayurveda, Traditional Chinese Medicine, and various indigenous healing practices have utilized botanicals such as *Garcinia indica* (Thouars) Choisy [Clusiaceae], *Curcuma longa* L. [Zingiberaceae] (Chandrasekaran et al., 2012), *Camellia sinensis* (L.) Kuntze [Theaceae] (Esteghamati et al., 2015), and *Ephedra sinica* Stapf [Ephedraceae] (Kim et al., 2014) for their purported ability to modulate appetite and support balanced body composition. However, the growing commercialization of herbal weight-loss products has led to the misrepresentation of traditional knowledge, with many formulations being adulterated with synthetic pharmaceuticals to amplify their effectiveness (Alyas et al., 2024).

The shift from traditional ethnomedicine to commercially available weight-loss supplements has been primarily driven by consumer demand and the prevalent belief that natural products are harmless (Ekor, 2014). This assumption, however, overlooks the complexity of herbal pharmacology, the potential for herb-drug interactions, and the significant risks associated with intentional adulteration (Aldewachi et al., 2020; Okoya et al., 2023; Wong et al., 2021). Ethnopharmacological investigations have revealed that while certain plants exhibit genuine anti-obesity effects (Chandrasekaran et al., 2012; Kim et al., 2014), many commercial formulations either lack sufficient bioactive compounds due to poor-quality sourcing or are deliberately enhanced with synthetic substances to produce rapid weight loss (Hamidi, 2023; Rocha et al., 2016). Such adulteration typically occurs through three primary mechanisms: (1) the illegal addition of synthetic pharmaceuticals - including anorectics (e.g., sibutramine), stimulants (e.g., ephedrine), and diuretics—to enhance weight-loss effects; (2) the substitution of high-value medicinal plants with lower-quality or botanically similar but less effective alternatives, thereby diminishing therapeutic efficacy; and (3) mislabeling or contamination, wherein products either lack the stated herbal ingredients or contain them in subtherapeutic doses, leading to inconsistent efficacy and potential safety concerns (Alyas et al., 2024).

This trend raises concerns about the erosion of traditional medical knowledge and the exploitation of herbal medicine for profit-driven motives (Alyas et al., 2024; Reyes-García et al., 2013). Furthermore, the presence of pharmaceutical adulterants undermines the integrity of traditional herbal practices and poses substantial health risks, particularly concerning product quality and safety (Aldewachi et al., 2020; Pittler and Ernst, 2004). In order to develop a long-term strategy to overcome the challenges of poor quality and adulterated products making medical claims, the aim of this systematic review and meta-analysis is to estimate the prevalence of pharmaceutical adulteration, particularly with sibutramine, in weight-loss natural products and to examine the characteristics of such adulterations.

## 2 Materials and methods

### 2.1 Search strategy

This systematic review and meta-analysis was performed following the guidelines outlined in the Cochrane Handbook and the Preferred Reporting Items for Systematic Reviews and Meta-Analyses (PRISMA). A systematic search was carried out across three electronic databases: PubMed, CINAHL, and Google Scholar to identify relevant publications on synthetic pharmaceutical adulterants in weight-loss natural products, covering studies from their inception to July 2024. Additionally, a manual search of reference lists from eligible publications was undertaken to identify relevant articles not indexed in the primary databases. The search strategy employed keywords and their synonyms across three main domains: “weight loss,” “adulterants,” and “natural products.” Initially, search terms were applied individually and subsequently combined using Boolean operators such as “AND” and “OR.”

The detailed search string used was: (weight loss OR slimming) AND (herbal products OR herbal supplements OR herbal remedies OR dietary supplements OR natural products OR food supplements OR herbal medicine) AND (adulteration OR synthetic pharmaceuticals OR contaminants OR chemical adulterants).

### 2.2 Selection criteria

Primary studies were eligible for inclusion if they were chemical-laboratory studies reporting the detection of synthetic pharmaceutical adulterants in weight-loss ‘herbal’ preparations, including herbal supplements, dietary supplements, herbal medicines, or food supplements, without language restrictions. Studies were excluded if they were review articles, case reports and conference papers. Additionally, studies with inadequate data to calculate the prevalence rate of adulteration were excluded from the quantitative analysis. Publications were managed using Endnote™ 21 (Clarivate Analytics, PA), which facilitated the importation of references and the removal of duplicate publications.

### 2.3 Data screening and extraction

Titles and abstracts of the identified studies were independently screened by two reviewers (DTAP and THV) to verify compliance with the selection criteria. Following this, a thorough review of the full texts was conducted for potentially eligible articles. For non-English articles, translations were conducted using Google Translate to facilitate evaluation (Jackson et al., 2019). Each researcher independently entered data into spreadsheets, capturing key attributes, including the author, year of publication, country, product information, formulation type, sample collection method, adulterants of interest, analytical methods, and the number of samples adulterated with synthetic pharmaceutical substances.

The adulteration prevalence rate was determined by dividing the number of adulterated samples by the total number of collected samples. Likewise, the prevalence of sibutramine adulteration was calculated as the proportion of samples containing sibutramine relative to the overall sample count.

### 2.4 Assessment of quality of study

Two reviewers (DTAP and TCMD) independently evaluated the quality of the included studies using the Toxicological Data Reliability Assessment Tool (ToxRTool), a standardized instrument for assessing the reliability of toxicological research (Schneider et al., 2009). The ToxRTool^®^ consists of two sections tailored for *in vivo* and *in vitro* studies. For this analysis, the *in vitro* section, comprising 18 criteria, was utilized as it was applicable to all included studies. Each criterion was assigned a score of 1 (“criterion met”) or 0 (“criterion not met”). The evaluation covered key aspects such as (i) identification of the test substance, (ii) characterization of the test system, (iii) description of the study design, (iv) documentation of study results, and (v) the plausibility of both the study design and findings. The total score determined the study’s quality category: category 1 (15–18 points) indicated good quality, category 2 (11–14 points) indicated moderate quality, and category 3 (<11 points) indicated low quality.

Any differences in data extraction or quality assessment were reconciled through agreement between the two reviewers (DTAP and TCMD) with additional investigators (CK and MH).

### 2.5 Data synthesis and statistical analysis

The overall prevalence of adulteration was estimated across the included studies using the DerSimonian-Laird random-effects model with corresponding 95% confidence intervals (CI) displayed in forest plots (DerSimonian and Laird, 1986). Study heterogeneity was evaluated through *χ* ^2^ tests and the I^2^ statistic (Borenstein et al., 2009). In cases of substantial heterogeneity (I^2^ ≥ 75%), prevalence estimates were summarized using the median and interquartile range (IQR). To identify potential sources of heterogeneity, subgroup analyses and univariate random-effects meta-regression were conducted. Publication bias was assessed using a funnel plot and Egger’s asymmetry test (Mavridis and Salanti, 2014). All statistical analyses were performed using STATA 17.0 (Stata Corp LLC, College Station, TX, United States), with a two-sided significance threshold of P < 0.05.

## 3 Results

### 3.1 Search outcomes

**FIGURE 1:**
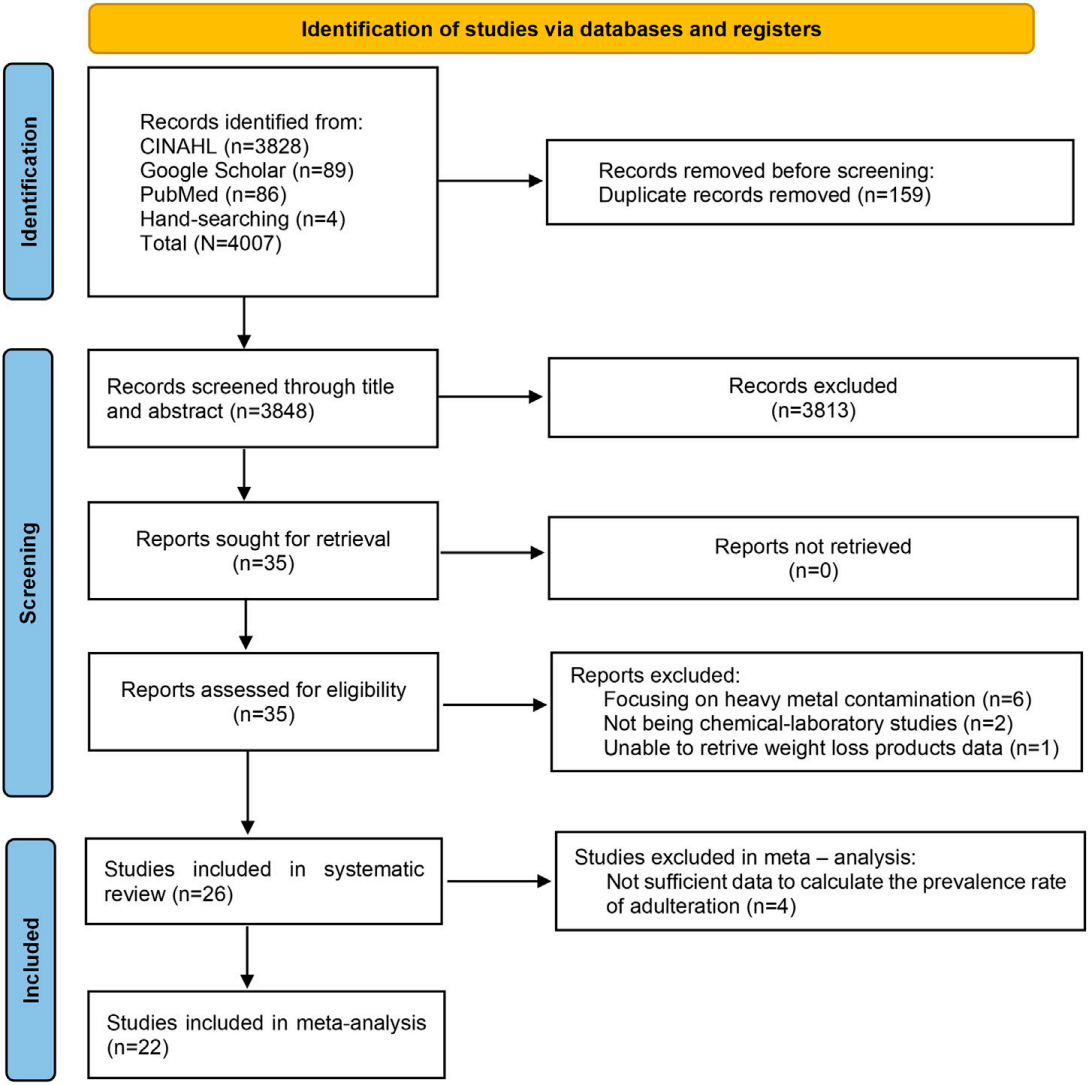
PRISMA flow diagram.

A total of 4,007 articles were identified, with 4,003 retrieved from database searches and 4 from manual searches. After duplicate removal, 3,848 articles remained for eligibility screening. Following title and abstract review, 3,813 articles were excluded. The remaining 35 articles underwent full-text assessment, leading to the exclusion of nine studies for the following reasons: focusing on heavy metal contamination (six articles), not being chemical-laboratory studies (two articles), and could not retrieve data on weight-loss products (one article). The final selection process is summarized in Figure 1.

### 3.2 Characteristics of included studies

A total of 26 studies were included in the systematic review (Table 1) published in English between 2012 and 2021. It includes nine studies conducted in Asia (35%) (Girish et al., 2020; Hoa et al., 2020; Jin et al., 2017; Karamahito et al., 2021; Phattanawasin et al., 2012; Saichanapan et al., 2020; Wong et al., 2021; Yun et al., 2018; Zhong et al., 2017), seven in Europe (27%) (Ancuceanu et al., 2013; Croitoru et al., 2017; Hachem et al., 2016; Mathon et al., 2014; Monakhova et al., 2012; Reeuwijk et al., 2014; Wu et al., 2020), six in Middle East (23%) (Dastjerdi et al., 2018; Firozian et al., 2021; Jairoun et al., 2021; Kamil, 2016; Khazan et al., 2014; Salahshour et al., 2020), three in the America (12%) (Carvalho et al., 2012; Moreira et al., 2013; Wilson and Masse, 2016) and one in Africa (Ahmed et al., 2019).

**TABLE 1:** Characteristics of studies investigating adulterants in natural weight-loss products.

Authors	Country	Products information	Formulation type	Sampling collection	Adulterants of interest	Adulterants identified in samples	Analytical
Samples collected from the internet							
Monakhova et al. (2012)	Russia	NR	Capsule, slimming tea	Weight-loss supplements were purchased directly by Internet mail	Caffeine	Caffeine: 40%	H-NMR
Mathon et al. (2014)	Switzerland	50 food supplements and two soluble beverages	Soluble dosage form	Food supplements and two soluble beverages were purchased on the Internet.	Sibutramine	NR	HPTLC-UV
Jin et al. (2017)	China	NR	Capsule, tea powder, tablet	Slimming supplements were purchased from a social networking platform	Fenfluramine, phenolphthalein, bumetanide, sibutramine	Fenfluramin: 14%	UPLC–MS/MS
Girish et al. (2020)	India	NR	NR	Different brands of weight loss products were purchased through web stores	Phenolphthalein, fluoxetine, sibutramine, nor-fenfluramine, ephedrine	No adulterants found	LC-ESI–MS/MS
Wu et al. (2020)	France	NR	NR	Products were bought on the Internet.	Phenolphthalein and sibutramine	Phenolphthalein: 23.1%	LF-NMR
Karamahito et al. (2021)	Thailand	Dietary supplements, slimming drink powder and slimming coffee powder	NR	Products were purchased through the internet Songkhla Province, Thailand	Sibutramine	Sibutramine: 37.5%	Distance-based paper device
Samples collected from local markets							
Khazan et al. (2014)	Iran	Magic Slim, Green lean Super Slim, Original Super Slim, Fast Slim, Herbaceous essence, Fat loss	Capsule, pill	Samples were obtained from the market	Sibutramine, phenolphthalein, phenytoin, bumetanide and rimonabant, caffeine, pseudoephedrine, theobromine and amfepramone	Sibutramine: 75%	GC-MS and LC-MS
Reeuwijk et al. (2014)	Netherlands	NR	NR	Herbal supplements for weight loss were sampled on the market.	Sibutramine, desmethylsibutramine, didesmethylsibutramine, phenolphthalein	Sibutramine: 34%	HPLC-DAD-MS/MS
Saichanapan et al. (2020)	Thailand	NR	NR	Products were purchased from the local market in Hat Yai, Thailand	Sibutramine	Sibutramine: 100%	Porous graphene ink-modified electrode (PGr-ink/GCE)
Samples collected from the pharmacies							
Carvalho et al. (2012)	Brazil	NR	Capsule	Herbal weight loss preparations were acquired from 73 pharmacies in nine Brazilian states	Amfepramone, fenproporex, sibutramine, fluoxetine, bupropion, sertraline, paroxetine and flurazepam	Fenproporex: 2.8%	Capillary electrophoresis
Ancuceanu et al. (2013)	Romania	NR	NR	Products (batch no. 290909 and 090203) were purchased from a community pharmacy	Sibutramine, phenolphthalein	NR	HPLC
Moreira et al. (2013)	Brazil	NR	NR	Samples were acquired from pharmacies in different regions of Brazil	Diuretics: furosemide, hydrochlorothiazide, amiloride and chlorthalidone	Hydrochlorothiazide: 23.1%	Capillary electrophoresis
Ahmed et al. (2019)	Egypt	Zotreem Plus® (MTCO Company, Hong Kong, China), Zotreem Extra® (MTCO Company), Malaysian Super Slim® (Majestic Group Australia P/L, Kuala Lumpur, Malaysia), AB Slim® (AB Care Medical Herbs, Beirut, Lebanon), Chinese Super Slim® (Yunnan Menghuang Trade Company, Hong Kong, China), and Metabolites® (Simildiet Laboratorios, Zaragoza, Spain)	NR	Products were purchased from different pharmacies in Alexandria, Egypt	Sibutramine, sildenafil, phenolphthalein, and orlistat	NR	HPLC
Hoa et al. (2020)	Vietnam	NR	Hard capsules, soft capsules and teabag	Samples were randomly collected from pharmacies in Hanoi, Vietnam	Sibutramine	Sibutramine: 20%	LC-MS/MS
Wong et al. (2021)	Australia	NR	Tablets or capsules	Samples were purchased from local pharmacies in Sydney, Australia	Sibutramine and caffeine	No adulterants found	Thin-layer chromatography
Samples collected from the stores (herbs, dietary supplements)							
Croitoru et al. (2017)	Romania	NR	Tea, capsule	Slimming capsules and teas were purchased from dedicated stores	Ephedrine, caffeine	Caffeine: 30%	HPLC
Salahshour et al. (2020)	Iran	NR	Tablets, capsules, powders, topical gels, and oral drops	Samples were gathered from herb shops	Caffeine, trimethoxyamphetamine, vitamin E	Caffeine: 21.8%	HPLC-DAD and GC-MS
Firozian et al. (2021)	Iran	NR	NR	Samples of available herbal weight loss products were randomly collected from local store in Hamadan	Sibutramine, methamphetamine	Sibutramine: 26.98%	HPLC
Samples collected from mix sources							
Phattanawasin et al. (2012)	Thailand	NR	Cachet, capsule, gel, coffee	Products of herbal slimming formulations were purchased from drugstores and via Internet.	Sibutramine	NR	Thin layer chromatography/densitometry
Hachem et al. (2016)	France	Food supplements were mainly formulated as conventional or softgel capsules, a few tablets, soluble coffee powders, other soluble powders, tea bags, drinkable liquid and patch	Soft gel capsule, tablet, soluble coffee powder, tea bag	Food supplements were bought mainly on internet web sites but also for a few numbers in specialized shops	Sibutramine, phenolphthalein, orlistat, lorcaserin, fluoxetine, sildnafil	Sibutramine: 26%	H-NMR
Wilson and Masse (2016)	USA	NR	Hard gelatin powder-filled capsules, tablets, and oil-filled soft gelatin capsules	Samples of dietary weight-loss supplements were obtained from local pharmacies and over the Internet.	Anorexics, anxiolytics, antidepressants, diuretics, laxatives, subutramine, N-desmethyl sibutramine and N-di-desmethyl sibutramine), orlistat, lorcaserin, phentermine, diethylpropion (amfepramone), fenfluramine, and rimonabant, caffeine and ephedrines	Sibutramine: 36.4%	LC-MS/MS
Dastjerdi et al. (2018)	Iran	NR	Tablets, handmade capsules, herbal teas, herbal jells and herbal resins	Herbal drugs advertised as weight reducing agents were acquired from herb shops or internet all over the Kermanshah province	Tramadol, Caffeine, Rizatriptan, Fluoxetine, Venlafaxine, Ritodrine, Without drug	Tramadol: 21.3%	GC/MS
Yun et al. (2018)	Korea	Dietary, herbal, and nutritional supplements, health functional foods, teas, and beverages advertised for weight-loss or fat-burning	Tablet, capsule, scoop, or teabag	Slimming foods advertised for weight-loss or fat-burning were purchased from local markets and on internet websites	Anorectics: sibutramine, orlistat, fenfluramine	NR	HPLC-PDA and UPLC-MS/MS

NR, not reported.

Regarding sampling locations, three studies did not specify the location of the sample (Jairoun et al., 2021; Kamil, 2016; Zhong et al., 2017). Out of 23 studies, six (26.1%) obtained samples randomly from community pharmacies (Ahmed et al., 2019; Ancuceanu et al., 2013; Carvalho et al., 2012; Hoa et al., 2020; Moreira et al., 2013; Wong et al., 2021). Six studies (21.6%) collected samples from the internet (Girish et al., 2020; Jin et al., 2017; Karamahito et al., 2021; Mathon et al., 2014; Monakhova et al., 2012; Wu et al., 2020). Three studies (13%) obtained samples from local markets (Khazan et al., 2014; Reeuwijk et al., 2014; Saichanapan et al., 2020) or herb/grocery stores (13%) (Croitoru et al., 2017; Firozian et al., 2021; Salahshour et al., 2020). The remaining five studies (21.7%) gathered samples from mixed sources such as pharmacies and internet, herb/grocery stores and internet or local markets and internet (Dastjerdi et al., 2018; Hachem et al., 2016; Phattanawasin et al., 2012; Wilson and Masse, 2016; Yun et al., 2018).

Various analytical techniques have been applied to detect adulteration. Among these, chromatographic methods are the most commonly used, accounting for 77% of studies (20/26 studies), including GS-MS, LC-MS, (high performance) thin-layer chromatography, HPTLC-UV, HPLC-DAD-MS/MS, HPLC, distance-based paper chromatography, LC-MS/MS, UPLC–MS/MS, LC-ESI–MS/MS, HPLC-DAD, HPLC-PDA, and RP-HPLC-MS/MS. Spectroscopic techniques, such as H-NMR and LF-NMR, were employed in 12% of studies (3/26 studies), while electrophoretic methods, including capillary electrophoresis and porous graphene ink-modified electrodes (PGr-ink/GCE), were also used in 12% of studies (3/26 studies).

Regarding the identified adulterants, a diverse range of synthetic and natural compounds has been detected, including stimulants (e.g., amphetamine, β-Methylphenethylamine [BMPEA], ephedrine, β-Phenylethylamine [β-PEA], yohimbine, caffeine), laxatives (e.g., sennosides, phenolphthalein), diuretics (e.g., furosemide, hydrochlorothiazide, amiloride, chlorthalidone), anorexiants (e.g., sibutramine, orlistat, fenfluramine), antidepressants (e.g., fluoxetine, paroxetine, sertraline, bupropion), and anxiolytics (e.g., diazepam, alprazolam, flurazepam, chlordiazepoxide, lorazepam, bromazepam, clonazepam). Among these substances, sibutramine was the most frequently identified (23/26 studies, 88.5%).

### 3.3 Assessment of quality of study

Using the ToxRTool for quality assessment, 17 out of the 26 included studies (59%) met most of the criteria and were categorized as high quality (Ancuceanu et al., 2013; Girish et al., 2020; Hachem et al., 2016; Hoa et al., 2020; Jairoun et al., 2021; Jin et al., 2017; Khazan et al., 2014; Mathon et al., 2014; Monakhova et al., 2012; Moreira et al., 2013; Phattanawasin et al., 2012; Reeuwijk et al., 2014; Saichanapan et al., 2020; Wilson and Masse, 2016; Wu et al., 2020; Yun et al., 2018; Zhong et al., 2017). Eight studies (28%) (Ahmed et al., 2019; Carvalho et al., 2012; Croitoru et al., 2017; Dastjerdi et al., 2018; Firozian et al., 2021; Karamahito et al., 2021; Salahshour et al., 2020; Wong et al., 2021) were rated as being of moderate quality. One study (3%) (Kamil, 2016) was classified as low quality.

### 3.4 The content of common synthetic adulterants in weight loss natural products

Among the 26 studies reviewed, 15 studies (58%) quantified the levels of adulterants detected, with sibutramine being the most frequently identified (12/15 studies, 80%) (Ahmed et al., 2019; Ancuceanu et al., 2013; Carvalho et al., 2012; Firozian et al., 2021; Hachem et al., 2016; Hoa et al., 2020; Karamahito et al., 2021; Khazan et al., 2014; Mathon et al., 2014; Monakhova et al., 2012; Reeuwijk et al., 2014; Yun et al., 2018), followed by phenolphthalein (5/15 studies, 33%) (Ahmed et al., 2019; Ancuceanu et al., 2013; Hachem et al., 2016; Khazan et al., 2014; Yun et al., 2018). The measurement units differed among studies. In five studies, the median concentration of sibutramine was 72.7 mg/g (IQR 16.1–97.3). Meanwhile, six other studies reported a median amount of 8.6 mg/capsule (IQR 2.7–17.8). One study identified adulteration levels ranging from 4–36 mg/day. Phenolphthalein was detected at a median level of 105.6 mg/g (IQR 22.0–189.8) and 51.58 mg/capsule (IQR 34.9–825.0).

### 3.5 Pooled prevalence of adulteration in weight loss natural products

**FIGURE 2:**
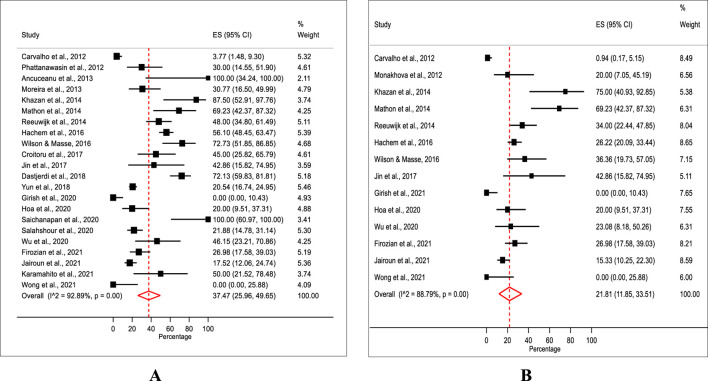
**(A)** Pooled prevalence of synthetic adulteration. **(B)** Pooled prevalence of sibutramine.

A total of 22 studies with sufficient data for estimating the prevalence of adulteration were included in the meta-analysis. A meta-analysis of 22 studies revealed a pooled prevalence rate of synthetic adulteration in weight-loss natural products with a median of 37.5% (IQR 25.9%–49.6%) with a significant heterogeneity (χ^2^ = 295.5, df = 21, P = 0.00, *I* ^2^ = 92.9%). The median prevalence rates showed substantial variability, ranging from 0% in studies conducted in India (0%, IQR 0.0%–10.4%) (Girish et al., 2020) and Australia (0%, 0.0%–25.9%) (Wong et al., 2021) to 100% in studies in Romania (100%, IQR 34.2%–100.0%) (Ancuceanu et al., 2013) and Thailand (100%, IQR 60.9%–100.0%) (Saichanapan et al., 2020) (Figure 2A).

In terms of sibutramine, the estimated prevalence of sibutramine adulteration in natural weight-loss products was found to have a median rate of 21.8% (IQR 11.9%–33.5%), with substantial heterogeneity (χ^2^ = 115.9, df = 13, P = 0.00, *I* ^2^ = 88.8%) (Figure 2B).

### 3.6 Subgroup analyses

From a geographical perspective, the highest prevalence rate of adulteration was observed in Europe, with a median of 54.7% (IQR 48.0%–61.4%), followed by the Middle East at 41.3% (IQR 19.4%–64.9%), America at 31.1% (IQR 0.3%–78.4%) and Asia at 24.7% (IQR 9.2%–43.8%).

In terms of publication period, more recent studies (publications during year 2020–2021) reported a lower median prevalence rate of adulteration at 23.3% (IQR 11.3%–37.7%) compared to studies published during 2016–2018 and 2012–2014, which documented median prevalence rates of 54.4% (IQR 28.2%–74.3%) and 45.9% (IQR 17.3%–75.9%), respectively.

The lowest prevalence of adulteration was observed in samples collected from pharmacies, with a median prevalence of 14.8% (IQR 0.44%–38.5%). In contrast, the highest prevalence was found in samples obtained from local markets, where the median prevalence was 79.4% (IQR 38.2%–100.00%). Samples purchased from the internet and general stores (such as herb or grocery stores) exhibited comparable prevalence rates, with medians of 35.8% (IQR 3.7%–76.4%) and 27.7% (IQR 17.8%–38.8%), respectively (Table 2).

**TABLE 2:** Subgroup analyses testing heterogeneity sources.

Characteristics	Studies (n)	Prevalence estimate % (95% CI)	Heterogeneity test			
			χ^2^	df	*P value*	*I* ^*2*^ (%)
Continent						
America	3	Median 31.1 (0.3–78.4)	53.5	2	*<0.001*	96.3
Asia	8	Median 24.7 (9.2–43.8)	52.3	7	*<0.001*	86.6
Europe	6	Median 54.7 (48.0–61.4)	4.9	5	*0.42*	0.00
Middle East	5	Median 41.3 (19.4–64.9)	71.8	4	*<0.001*	94.4
Publication period						
Year 2012–2014	7	Median 45.9 (17.3–75.9)	84.9	6	*<0.001*	92.9
Year 2016–2018	6	Median 54.4 (28.2–74.3)	117.10	5	*<0.001*	95.7
Year 2020–2021	9	Median 23.3 (11.3–37.7)	55.9	8	*<0.001*	85.7
Sampling location						
Pharmacies	5	Median 14.8 (0.4–38.5)	26.8	4	*<0.001*	85.1
Mix locations	6	Median 43.5 (23.8–64.3)	135.9	5	*<0.001*	96.3
Local market	3	Median 79.4 (38.2–100)	11.6	2	*<0.001*	82.8
Internet	5	Median 35.8 (3.7–76.4)	42.4	4	*<0.001*	90.6
Herb/grocery store	3	Median 27.7 (17.8–38.8)	4.19	2	*0.12*	52.3

### 3.7 Meta-regression and publication bias

Meta-regression analyses were performed to explore potential contributors to heterogeneity in the pooled prevalence of synthetic adulteration. The analysis examined covariates such as geographic region, publication period, and sampling source. However, none of these factors showed a statistically significant relationship with the overall prevalence of adulteration (P > 0.05) (Table 3).

**TABLE 3:** The potential factors contributing to heterogeneity in the prevalence of adulteration using univariate meta-regression analysis.

Factors	Coef	Std. Err	t_21_ (95% CI)	*P value*
Continent	0.054	0.053	1.03 (−0.055, 0.164)	*0.313*
Publication period	−0.079	0.069	−1.15 (−0.225, 0.065)	*0.264*
Sampling location	0.021	0.042	0.50 (−0.067,0.109)	*0.620*

**FIGURE 3:**
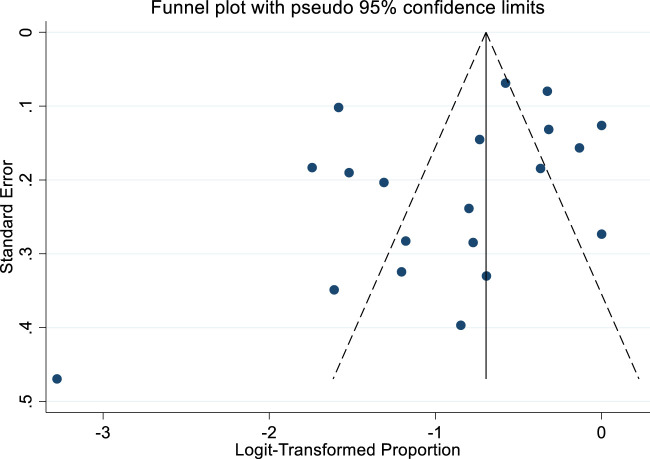
Bias assessment funnel plot of studies reported adulteration in weight loss natural products.

The funnel plot analysis (Figure 3) showed no indication of publication bias, as evidenced by its symmetrical distribution. Furthermore, Egger’s test did not detect any significant small-study effects (P = 0.153).

## 4 Discussion

Due to side effects associated with synthetic drugs, there is a growing interest in alternative therapies, including natural products (herbal medicines and nutraceuticals/botanicals) for weight management (Bahmani et al., 2016). As these products become increasingly popular, the adulteration of these products with undeclared synthetic substances has become a significant concern for drug regulatory authorities, given the potential health risks involved (Calahan et al., 2016). This systematic review and meta-analysis highlighted that around one-third weight-loss natural products were adulterated by synthetic drugs based on chemical laboratory-studies. The highest occurrence was reported in Europe at 54.7% (IQR 48.0%–61.4%), with low heterogeneity (*I* ^*2*^ = 0.00, P = 0.42), where sibutramine was the most detected adulterant. Obesity has emerged as a global epidemic, increasingly affecting Europe and numerous fast developing economies (Barness et al., 2007; Koncz et al., 2020). Our findings are consistent with prior analyses, such as the Rapid Alert System for Food and Feed (RASFF) database review, which reported unauthorized synthetic weight-loss agents in 63.3% of products with quality concerns (Koncz et al., 2020).

Classified as over-the-counter products, weight-loss preparations are readily accessible to consumers, often from sources with questionable quality standards. Many individuals may unknowingly consume products adulterated with varying amounts of approved prescription drugs, untested pharmaceutical ingredients, or other active substances that lack proper evaluation (Biesterbos et al., 2019; Tucker et al., 2018). This raises important questions about the need for a medical regulation of such products *in lieu* of the commonly used regulation as a food supplement/botanical or not systematically regulating these products at all (Heinrich, 2015). Based on the studies reviewed, product samples were obtained from diverse sources, including community pharmacies, local markets, herb/grocery stores, and online platforms. Subgroup analysis revealed the highest prevalence of adulteration in products sourced from local markets (79.4%), followed by internet purchases (35.8%) and herb/grocery stores (27.7%) whereas samples from community pharmacies exhibited a lower adulteration rate (14.8%). This scenario presents considerable risks to consumers and highlights the varying levels of regulatory oversight across distribution channels. Most evidence published in peer-reviewed journals indicates that weight-loss dietary supplements have limited effectiveness and are frequently linked to significant risks, such as allergic reactions and drug interactions when used for extended periods without medical oversight (Aldewachi et al., 2020; Pittler and Ernst, 2004). Adverse effects can arise from the pharmacological actions of the adulterants or interactions with other drugs or herbs, even at concentrations lower than those found in prescribed pharmaceuticals (Dastjerdi et al., 2018; de Carvalho et al., 2011). Moreover, the financial burden of purchasing these products can be particularly substantial for low-income people with estimates indicating that consumers collectively spend over $20 billion annually on such products (Wheatley and Spink, 2013).

To effectively protect public health from adulteration in weight-loss natural products, a multi-faceted approach is essential. Considering the substantial prevalence of adulteration, estimated at 37.5% (IQR 25.9%–49.6%), along with its limited efficacy and potential health risks (Pittler and Ernst, 2004), dietary supplements for weight loss should not be recommended, especially when safer and more effective alternatives such as modern lifestyle modifications, pharmacological therapies, and surgical interventions are available (Heymsfield, 2023; Manore and Patton-Lopez, 2022). On the other hand, as long as these products remain widely used, ensuring their quality, safety, and efficacy through a comprehensive global framework is crucial. First, global regulatory standards must be aligned with guidelines from authoritative organizations such as the World Health Organization (1998), World Health Organization (2004) and major pharmacopoeias, such as the United States Pharmacopeia (Upton et al., 2011) and European Pharmacopoeia. Harmonized standards will help reduce discrepancies between countries, promoting consistency in product quality. Comprehensive labeling regulations should mandate the disclosure of ingredient sourcing, production methods, and potential contaminants, fostering transparency across the supply chain (Food and Agriculture Organization, 2009). Second, robust surveillance systems are essential for detecting and reporting adulteration, supported by legal frameworks that impose stringent penalties to deter violations (Ariffin et al., 2021; Manning and Soon, 2014). Addressing adulteration on a global scale requires enhanced cross-border cooperation and information sharing. A first and essential step could be a global database of reported adulteration cases and verified natural products providing regulators, patient groups, industry stakeholders and researchers, with reliable data to support their efforts (Manning and Soon, 2014). Third, educating consumers about the risks associated with adulterated products is essential for promoting informed purchasing decisions. Public awareness campaigns can highlight the importance of buying from reputable sources and recognizing signs of product adulteration (Hamidi, 2023; Jairoun et al., 2021; Kongkaew et al., 2024). Additionally, training healthcare professionals - including pharmacists, clinicians, and traditional medicine practitioners - to identify and report suspicious products will help protect public health (Cohen, 2018; Conway et al., 2023).

Future research should prioritize the development of predictive models to assess the risk of adulteration in specific products or regions, enabling proactive risk management. Additionally, integrating these models with the design and development of affordable and user-friendly test kits for detecting specified adulterants (such as sibutramine and phenolphthalein) could empower consumers to self-check products, reducing reliance on healthcare professionals and mitigating public health risks (Manning and Soon, 2014). Furthermore, evaluating the socioeconomic impact of adulteration is critical for understanding its broader implications on public health systems, traditional medicine markets, and consumer confidence. Research should assess the effectiveness of existing regulations, identifying barriers to enforcement and proposing solutions for more efficient global governance.

### 4.1 Strengths and limitations

This study has several strengths, such as a comprehensive literature search without language restrictions, a dual data extraction process, and a rigorous evaluation of study quality. Nevertheless, there are also limitations. First, due to a lack of sufficient information, we were unable to gather detailed data on weight-loss products such as brands, origins, and herbal components. Such information provides essential insights for healthcare providers and regulatory agencies in overseeing the importation and supply of these products, particularly given that preparations originating from Asia have been reported as the most adulterated (Posadzki et al., 2013). Future studies should aim to provide as much detail as possible to offer useful information to the public. Second, we do not address unintentional adulteration, and this will certainly add to the risks to patients and consumers. Third, the included studies lacked detailed information on the sampling technique, making it possible that the sampling was not random in some cases which may have contributed to the high heterogeneity in the prevalence rate. Therefore, the results should be interpreted with caution. Future systematic review and meta-analysis on this topic could utilize more comprehensive databases - such as Scopus, Web of Science, Embase and/or OVID - to address the limitations identified in this study.

## 5 Conclusion

This systematic review and meta-analysis provides evidence that synthetic adulteration remains a pervasive issue in weight-loss natural products, with approximately one-third of products tested containing undeclared pharmaceutical agents. The high prevalence of adulteration, particularly in unregulated markets, underscores the urgent need for strengthened regulatory measures and enhanced consumer awareness. To safeguard public health and market integrity, a globally coordinated approach is essential, focusing on standardized regulations, international collaboration, and public awareness. Future research should assess long-term health effects to ensure sustainable and safe healthcare solutions worldwide.

## Data Availability

The original contributions presented in the study are included in the article/supplementary material, further inquiries can be directed to the corresponding author.
